# 2,2′-(Propane-2,2-di­yl)bis­(1*H*-pyrrole)

**DOI:** 10.1107/S1600536809054701

**Published:** 2010-01-16

**Authors:** Guillaume Journot, Reinhard Neier, Helen Stoeckli-Evans

**Affiliations:** a Institute of Chemistry, University of Neuchâtel, rue Emile-Argand 11, 2009 Neuchâtel, Switzerland; b Institute of Physics, University of Neuchâtel, rue Emile-Argand 11, 2009 Neuchâtel, Switzerland

## Abstract

The title compound, C_11_H_14_N_2_, crystallized with two independent mol­ecules (*A* and *B*) in the asymmetric unit. The two mol­ecules differ only slightly, with the pyrrole rings being inclined to one another at a dihedral angle of 87.67 (8)° in mol­ecule *A* and 88.09 (7)° in mol­ecule *B*. In the crystal, there are no classical hydrogen bonds, but the two pyrrole NH groups of one mol­ecule are involved in N—H⋯π inter­actions with the pyrrole rings of the other mol­ecule. In this manner, a compact box-like arrangement of the two independent mol­ecules is formed.

## Related literature

For substituted calix[4]pyrroles, see: Gale *et al.* (1998 ▶); Sessler & Davis (2001 ▶); Sessler *et al.* (2003 ▶). For the synthesis and crystal structure of *meso*-diethyl-bis­(2-pyrrol­yl)methane, see: Sobral *et al.* (2003 ▶). For inter­molecular inter­actions involving aromatic rings in biological systems, see: Meyer *et al.* (2003 ▶). For a spectroscopic analysis of N—H⋯π inter­actions in pyrroles, see: Dauster *et al.* (2008 ▶).
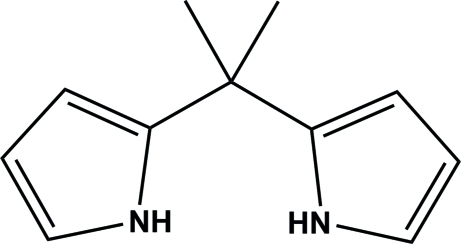


## Experimental

### 

#### Crystal data

C_11_H_14_N_2_
*M* *_r_* = 174.24
Triclinic, 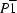

*a* = 8.4554 (8) Å
*b* = 9.2001 (8) Å
*c* = 13.2274 (11) Å
α = 99.802 (7)°
β = 95.321 (7)°
γ = 97.328 (7)°
*V* = 998.74 (15) Å^3^
*Z* = 4
Mo *K*α radiation
μ = 0.07 mm^−1^
*T* = 173 K
0.40 × 0.34 × 0.28 mm

#### Data collection

Stoe IPDS-2 diffractometer
15270 measured reflections
5385 independent reflections
3816 reflections with *I* > 2σ(*I*)
*R*_int_ = 0.046

#### Refinement

*R*[*F* ^2^ > 2σ(*F* ^2^)] = 0.045
*wR*(*F* ^2^) = 0.106
*S* = 1.02
5385 reflections
255 parameters
H atoms treated by a mixture of independent and constrained refinement
Δρ_max_ = 0.25 e Å^−3^
Δρ_min_ = −0.21 e Å^−3^

### 

Data collection: *X-AREA* (Stoe & Cie, 2009 ▶); cell refinement: *X-AREA*; data reduction: *X-RED32* (Stoe & Cie, 2009 ▶); program(s) used to solve structure: *SHELXS97* (Sheldrick, 2008 ▶); program(s) used to refine structure: *SHELXL97* (Sheldrick, 2008 ▶); molecular graphics: *ORTEP-3* (Farrugia, 1997 ▶) and *Mercury* (Macrae *et al.*, 2006 ▶); software used to prepare material for publication: *SHELXL97* and *PLATON* (Spek, 2009 ▶).

## Supplementary Material

Crystal structure: contains datablocks I, global. DOI: 10.1107/S1600536809054701/is2506sup1.cif

Structure factors: contains datablocks I. DOI: 10.1107/S1600536809054701/is2506Isup2.hkl

Additional supplementary materials:  crystallographic information; 3D view; checkCIF report

## Figures and Tables

**Table 1:** Geometry of N—H⋯π inter­actions (Å, °)

*D*	H	*Centroid*	N—H	H⋯*Cg*	*D*⋯*Cg*	N—H⋯*Cg*
N1	H1*N*	*Cg*4	0.86 (2)	2.534 (17)	3.2190 (12)	137.4 (14)
N2	H2*N*	*Cg*3	0.86 (2)	2.591 (17)	3.2425 (12)	133.7 (13)
N21	H21*N*	*Cg*1	0.88 (2)	2.523 (16)	3.1925 (12)	133.9 (12)
N22	H22*N*	*Cg*2	0.86 (2)	2.610 (17)	3.2440 (12)	131.3 (13)

*Cg*1, *Cg*2, *Cg*3 and *Cg*4 are the centroids of the N1/C1–C4, N2/C5–C8, N21/C21—C24 and N22/C25–C28 rings, respectively.

**Fig. 1.**
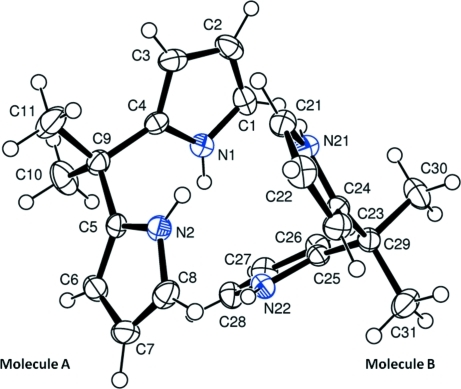
A view of the molecular structure of the two independent molecules (A and B) of the title compound, with the displacement ellipsoids drawn at the 50% probability level.

**Fig. 2.**
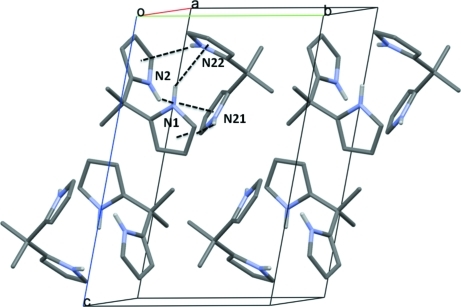
A view, along the a axis, of the crystal packing of the title compound. The N—H···π interactions are shown as dotted black lines for one of the box-like arrangements of the two independent molecules (see Table 1 for details; C-bound H-atoms have been omitted for clarity).

## supplementary crystallographic information

### Comment

The title compound was prepared as a building block for the formation of substituted calix[4]pyrroles. The latter have been shown to form extremely interesting host–guest complexes with various anions (Gale *et al.*, 1998; Sessler & Davis, 2001; Sessler *et al.*, 2003).

The structure of the title compound is shown in Fig. 1, and the geometrical parameters are given in the Supplementary Information and the archived CIF. The compound crystallized in the centrosymmetric triclinic space group *P*1 with two independent molecules (A and B) in the asymmetric unit. The bond lengths and angles are similar to those observed in the diethyl analogue (Sobral *et al.*, 2003), which also crystallized with two independent molecules, but in the non-centrosymetric monoclinic space group *C*2.

In the title compound the quateranry centers, C9 in A and C29 in B, impose a twist to the molecules with the pyrrole ring mean-planes being almost perpendicular to one another; 87.67 (8) ° in molecule A and 88.09 (7)° in molecule B. This is similar to the situation in the diethyl analogue where the two dihedral angles are 86.5 (2) and 86.7 (2) °.

N—H···π interactions are extremely important in biological systems and this aspect as been reviewed by (Meyer *et al.*, 2003). The spectroscopic aspects of the N—H···π interactions of the pyrrole dimer have also been studied recently by (Dauster *et al.*, 2008). In the crystal of the title compound the two independent molecules are linked by N—H···π interactions involving the pyrrole NH H-atoms of molecule A with the pyrrole rings of molecule B, and visa-*versa* (Table 1). This leads to the formation of a compact box-like arrangement of the two molecules, as shown in Fig. 2. Again this arrangement is similar to that observed in the crystal of the diethyl analogue.

### Experimental

A mixture of acetone (4.21 ml, 57.4 mmol) and pyrrole (31.72 ml, 0.459 mol, 8 equiv.) were stirred for 5 min and then trifluoroactetic acid (TFA: 0.44 ml, 2.53 mmol, 0.1 equiv) was added. The mixture stirred for an additional 5 min and then quenched with aqueous NaOH (0.1 N, 30 ml). It was then extracted with CH_2_Cl_2_ (50 ml × 2) and the organic layer dried (Na_2_SO_4_). The solvent was removed *in vacuo* and the remaining oil (82% pure in GC) was purified by flash chromatography on silica (eluent: cyclohexane/ethyl acetate; *v*:*v* = 4:1) to give colourless block-like crystals of the title compound (yield 6.8 g, 68%). ^1^H NMR (CDCl_3_): δ 7.72 (bs, 2H, N—H), 6.62–6.60 (m, 2H, pyrrolic-H1), 6.15–6.13 (m, 2H, pyrrolic-H2), 6.11–6.09 (m, 2H, pyrrolic-H3), 1.59 (s, 6H, –CH_3_); ^13^CNMR (CDCl_3_): δ 139.24 (C4), 117.19 (C1), 107.91 (C2), 103.87 (C3), 35.52(C5), 29.46 (C6).

### Refinement

The NH H-atoms were located in a difference electron-density map and were freely refined: N—H = 0.86 (2)–0.88 (2) Å. The C-bound H-atoms were included in calculated positions and treated as riding atoms: C—H = 0.95 and 0.99 Å for CH and CH_3_ H-atoms, respectively, with *U*_iso_(H) = k × *U*_eq_(C), where k = 1.2 for CH H-atoms, and 1.5 for CH_3_ H-atoms.

### Crystal data

**Table:**

C_11_H_14_N_2_	*Z* = 4
*M**_r_* = 174.24	*F*(000) = 376
Triclinic, *P*1	*D*_x_ = 1.159 Mg m^−^^3^
Hall symbol: -P 1	Mo *K*α radiation, λ = 0.71073 Å
*a* = 8.4554 (8) Å	Cell parameters from 10442 reflections
*b* = 9.2001 (8) Å	θ = 1.6–29.5°
*c* = 13.2274 (11) Å	µ = 0.07 mm^−^^1^
α = 99.802 (7)°	*T* = 173 K
β = 95.321 (7)°	Block, colourless
γ = 97.328 (7)°	0.40 × 0.34 × 0.28 mm
*V* = 998.74 (15) Å^3^	

### Data collection

**Table:**

Stoe IPDS-2 diffractometer	3816 reflections with *I* > 2σ(*I*)
Radiation source: fine-focus sealed tube	*R*_int_ = 0.046
graphite	θ_max_ = 29.2°, θ_min_ = 1.6°
φ and ω scans	*h* = −11→11
15270 measured reflections	*k* = −12→12
5385 independent reflections	*l* = −18→18

### Refinement

**Table:**

Refinement on *F*^2^	Primary atom site location: structure-invariant direct methods
Least-squares matrix: full	Secondary atom site location: difference Fourier map
*R*[*F*^2^ > 2σ(*F*^2^)] = 0.045	Hydrogen site location: inferred from neighbouring sites
*wR*(*F*^2^) = 0.106	H atoms treated by a mixture of independent and constrained refinement
*S* = 1.02	*w* = 1/[σ^2^(*F*_o_^2^) + (0.0549*P*)^2^ + 0.0012*P*] where *P* = (*F*_o_^2^ + 2*F*_c_^2^)/3
5385 reflections	(Δ/σ)_max_ < 0.001
255 parameters	Δρ_max_ = 0.25 e Å^−^^3^
0 restraints	Δρ_min_ = −0.21 e Å^−^^3^

### Special details

**Table:**

Geometry. Bond distances, angles *etc*. have been calculated using the rounded fractional coordinates. All su's are estimated from the variances of the (full) variance-covariance matrix. The cell e.s.d.'s are taken into account in the estimation of distances, angles and torsion angles
Refinement. Refinement of *F*^2^ against ALL reflections. The weighted *R*-factor *wR* and goodness of fit *S* are based on *F*^2^, conventional *R*-factors *R* are based on *F*, with *F* set to zero for negative *F*^2^. The threshold expression of *F*^2^ > σ(*F*^2^) is used only for calculating *R*-factors(gt) *etc*. and is not relevant to the choice of reflections for refinement. *R*-factors based on *F*^2^ are statistically about twice as large as those based on *F*, and *R*- factors based on ALL data will be even larger.

### Fractional atomic coordinates and isotropic or equivalent isotropic displacement parameters (Å^2^)

**Table:**

	*x*	*y*	*z*	*U*_iso_*/*U*_eq_	
N1	0.12135 (12)	0.25140 (11)	0.31284 (8)	0.0249 (3)	
N2	0.40689 (12)	0.01811 (11)	0.24234 (8)	0.0252 (3)	
C1	0.13663 (14)	0.38021 (14)	0.38492 (10)	0.0312 (4)	
C2	0.17507 (15)	0.34619 (16)	0.47962 (10)	0.0348 (4)	
C3	0.18351 (15)	0.19174 (16)	0.46471 (9)	0.0311 (4)	
C4	0.14934 (13)	0.13454 (13)	0.36055 (9)	0.0234 (3)	
C5	0.24635 (14)	−0.03734 (12)	0.22005 (9)	0.0226 (3)	
C6	0.22365 (16)	−0.10944 (14)	0.11865 (9)	0.0305 (4)	
C7	0.37369 (18)	−0.09563 (15)	0.07953 (10)	0.0358 (4)	
C8	0.48462 (16)	−0.01599 (14)	0.15732 (10)	0.0314 (4)	
C9	0.13255 (15)	−0.02364 (14)	0.30167 (9)	0.0277 (3)	
C10	−0.04155 (17)	−0.07156 (17)	0.24923 (13)	0.0470 (5)	
C11	0.1699 (2)	−0.12827 (17)	0.37732 (13)	0.0479 (5)	
N21	0.51399 (12)	0.37264 (11)	0.36601 (7)	0.0232 (3)	
N22	0.32603 (13)	0.28069 (11)	0.11006 (7)	0.0253 (3)	
C21	0.59997 (14)	0.28840 (14)	0.42014 (9)	0.0264 (3)	
C22	0.73108 (15)	0.26137 (15)	0.37047 (10)	0.0308 (4)	
C23	0.72320 (14)	0.33192 (14)	0.28313 (9)	0.0281 (4)	
C24	0.58798 (13)	0.40056 (13)	0.28184 (8)	0.0225 (3)	
C25	0.35755 (14)	0.42785 (13)	0.15697 (8)	0.0229 (3)	
C26	0.21946 (16)	0.48825 (14)	0.13876 (9)	0.0298 (4)	
C27	0.10133 (16)	0.37382 (15)	0.07998 (10)	0.0340 (4)	
C28	0.17016 (15)	0.24721 (14)	0.06366 (9)	0.0307 (3)	
C29	0.52308 (14)	0.49773 (13)	0.21071 (9)	0.0251 (3)	
C30	0.51173 (19)	0.65164 (14)	0.27414 (11)	0.0381 (4)	
C31	0.63878 (17)	0.51784 (17)	0.12898 (10)	0.0381 (4)	
H1N	0.1032 (19)	0.2477 (18)	0.2475 (13)	0.040 (4)*	
H2N	0.4531 (19)	0.0726 (18)	0.2990 (12)	0.040 (4)*	
H1	0.12280	0.47620	0.37120	0.0370*	
H2	0.19290	0.41380	0.54380	0.0420*	
H3	0.20840	0.13690	0.51730	0.0370*	
H6	0.12470	−0.15960	0.08150	0.0370*	
H7	0.39370	−0.13470	0.01140	0.0430*	
H8	0.59580	0.01080	0.15310	0.0380*	
H10A	−0.11590	−0.06340	0.30180	0.0710*	
H10B	−0.05400	−0.17510	0.21260	0.0710*	
H10C	−0.06530	−0.00660	0.20000	0.0710*	
H11A	0.09550	−0.12180	0.43000	0.0720*	
H11B	0.28040	−0.09850	0.41070	0.0720*	
H11C	0.15750	−0.23100	0.33940	0.0720*	
H21N	0.4231 (19)	0.4002 (16)	0.3827 (11)	0.031 (4)*	
H22N	0.3894 (19)	0.2146 (18)	0.1123 (11)	0.039 (4)*	
H21	0.57330	0.25470	0.48130	0.0320*	
H22	0.81210	0.20590	0.39070	0.0370*	
H23	0.79840	0.33180	0.23400	0.0340*	
H26	0.20540	0.58890	0.16150	0.0360*	
H27	−0.00570	0.38380	0.05650	0.0410*	
H28	0.11950	0.15250	0.02670	0.0370*	
H30A	0.47170	0.71520	0.22810	0.0570*	
H30B	0.61830	0.69770	0.30870	0.0570*	
H30C	0.43790	0.64010	0.32610	0.0570*	
H31A	0.64370	0.42070	0.08640	0.0570*	
H31B	0.74620	0.56090	0.16360	0.0570*	
H31C	0.60000	0.58470	0.08510	0.0570*	

### Atomic displacement parameters (Å^2^)

**Table:**

	*U*^11^	*U*^22^	*U*^33^	*U*^12^	*U*^13^	*U*^23^
N1	0.0216 (5)	0.0271 (5)	0.0262 (5)	0.0050 (4)	0.0023 (4)	0.0045 (4)
N2	0.0249 (5)	0.0242 (5)	0.0266 (5)	0.0033 (4)	0.0039 (4)	0.0051 (4)
C1	0.0204 (6)	0.0265 (6)	0.0442 (7)	0.0037 (5)	0.0068 (5)	−0.0019 (5)
C2	0.0263 (6)	0.0400 (7)	0.0321 (6)	−0.0005 (5)	0.0080 (5)	−0.0090 (5)
C3	0.0269 (6)	0.0430 (8)	0.0231 (6)	0.0032 (5)	0.0050 (4)	0.0058 (5)
C4	0.0187 (5)	0.0285 (6)	0.0244 (5)	0.0045 (4)	0.0055 (4)	0.0068 (4)
C5	0.0244 (5)	0.0184 (5)	0.0258 (5)	0.0029 (4)	0.0035 (4)	0.0059 (4)
C6	0.0395 (7)	0.0234 (6)	0.0267 (6)	0.0030 (5)	0.0002 (5)	0.0024 (5)
C7	0.0553 (9)	0.0276 (7)	0.0283 (6)	0.0115 (6)	0.0169 (6)	0.0051 (5)
C8	0.0332 (7)	0.0269 (6)	0.0402 (7)	0.0104 (5)	0.0174 (5)	0.0116 (5)
C9	0.0270 (6)	0.0264 (6)	0.0312 (6)	0.0027 (5)	0.0094 (5)	0.0071 (5)
C10	0.0282 (7)	0.0395 (8)	0.0639 (10)	−0.0074 (6)	0.0111 (6)	−0.0107 (7)
C11	0.0672 (11)	0.0390 (8)	0.0518 (9)	0.0195 (7)	0.0325 (8)	0.0256 (7)
N21	0.0186 (5)	0.0288 (5)	0.0227 (5)	0.0038 (4)	0.0016 (4)	0.0060 (4)
N22	0.0289 (5)	0.0225 (5)	0.0239 (5)	0.0058 (4)	−0.0029 (4)	0.0043 (4)
C21	0.0248 (6)	0.0302 (6)	0.0236 (5)	0.0013 (5)	−0.0040 (4)	0.0087 (5)
C22	0.0238 (6)	0.0332 (7)	0.0360 (7)	0.0079 (5)	−0.0029 (5)	0.0085 (5)
C23	0.0211 (6)	0.0336 (7)	0.0292 (6)	0.0037 (5)	0.0037 (4)	0.0047 (5)
C24	0.0211 (5)	0.0237 (6)	0.0209 (5)	0.0000 (4)	−0.0003 (4)	0.0034 (4)
C25	0.0283 (6)	0.0221 (6)	0.0186 (5)	0.0039 (4)	0.0003 (4)	0.0055 (4)
C26	0.0349 (7)	0.0250 (6)	0.0308 (6)	0.0094 (5)	−0.0012 (5)	0.0074 (5)
C27	0.0289 (6)	0.0382 (7)	0.0347 (7)	0.0056 (5)	−0.0074 (5)	0.0115 (6)
C28	0.0317 (6)	0.0303 (6)	0.0267 (6)	−0.0010 (5)	−0.0080 (5)	0.0063 (5)
C29	0.0275 (6)	0.0242 (6)	0.0223 (5)	0.0002 (4)	−0.0014 (4)	0.0057 (4)
C30	0.0508 (8)	0.0234 (6)	0.0354 (7)	0.0030 (6)	−0.0115 (6)	0.0029 (5)
C31	0.0351 (7)	0.0476 (8)	0.0315 (6)	−0.0054 (6)	0.0023 (5)	0.0163 (6)

### Geometric parameters (Å, °)

**Table:**

N1—C1	1.3724 (17)	C10—H10C	0.9800
N1—C4	1.3705 (16)	C10—H10B	0.9800
N2—C5	1.3739 (16)	C10—H10A	0.9800
N2—C8	1.3655 (17)	C11—H11B	0.9800
N1—H1N	0.858 (17)	C11—H11A	0.9800
N2—H2N	0.856 (16)	C11—H11C	0.9800
N21—C21	1.3704 (16)	C21—C22	1.3683 (18)
N21—C24	1.3720 (14)	C22—C23	1.4186 (18)
N22—C25	1.3712 (15)	C23—C24	1.3750 (17)
N22—C28	1.3750 (17)	C24—C29	1.5170 (16)
N21—H21N	0.875 (16)	C25—C29	1.5192 (17)
N22—H22N	0.863 (16)	C25—C26	1.3744 (18)
C1—C2	1.3626 (19)	C26—C27	1.4211 (19)
C2—C3	1.413 (2)	C27—C28	1.3612 (19)
C3—C4	1.3781 (17)	C29—C31	1.5421 (18)
C4—C9	1.5118 (17)	C29—C30	1.5373 (18)
C5—C6	1.3757 (17)	C21—H21	0.9500
C5—C9	1.5115 (17)	C22—H22	0.9500
C6—C7	1.413 (2)	C23—H23	0.9500
C7—C8	1.3654 (19)	C26—H26	0.9500
C9—C11	1.541 (2)	C27—H27	0.9500
C9—C10	1.544 (2)	C28—H28	0.9500
C1—H1	0.9500	C30—H30A	0.9800
C2—H2	0.9500	C30—H30B	0.9800
C3—H3	0.9500	C30—H30C	0.9800
C6—H6	0.9500	C31—H31A	0.9800
C7—H7	0.9500	C31—H31B	0.9800
C8—H8	0.9500	C31—H31C	0.9800
			
C1—N1—C4	109.93 (10)	C9—C11—H11C	109.00
C5—N2—C8	110.24 (10)	H11A—C11—H11B	109.00
C1—N1—H1N	123.9 (11)	H11A—C11—H11C	109.00
C4—N1—H1N	126.1 (11)	H11B—C11—H11C	109.00
C5—N2—H2N	126.5 (11)	C9—C11—H11B	109.00
C8—N2—H2N	123.1 (11)	C9—C11—H11A	109.00
C21—N21—C24	110.16 (10)	N21—C21—C22	107.86 (11)
C25—N22—C28	110.04 (10)	C21—C22—C23	107.02 (11)
C21—N21—H21N	123.5 (9)	C22—C23—C24	108.20 (11)
C24—N21—H21N	126.3 (9)	C23—C24—C29	131.63 (10)
C28—N22—H22N	123.0 (11)	N21—C24—C23	106.75 (10)
C25—N22—H22N	126.8 (11)	N21—C24—C29	121.55 (10)
N1—C1—C2	107.93 (12)	N22—C25—C29	121.59 (11)
C1—C2—C3	107.31 (12)	C26—C25—C29	131.53 (11)
C2—C3—C4	108.08 (11)	N22—C25—C26	106.79 (10)
C3—C4—C9	130.99 (12)	C25—C26—C27	108.09 (11)
N1—C4—C9	122.17 (10)	C26—C27—C28	107.19 (12)
N1—C4—C3	106.75 (11)	N22—C28—C27	107.89 (11)
N2—C5—C6	106.70 (11)	C25—C29—C31	109.40 (10)
C6—C5—C9	131.40 (11)	C30—C29—C31	108.82 (11)
N2—C5—C9	121.74 (10)	C25—C29—C30	109.04 (10)
C5—C6—C7	107.85 (11)	C24—C29—C25	110.94 (10)
C6—C7—C8	107.59 (12)	C24—C29—C30	109.35 (10)
N2—C8—C7	107.61 (12)	C24—C29—C31	109.26 (10)
C4—C9—C5	111.44 (10)	N21—C21—H21	126.00
C10—C9—C11	109.12 (12)	C22—C21—H21	126.00
C5—C9—C10	109.09 (10)	C21—C22—H22	126.00
C5—C9—C11	108.66 (11)	C23—C22—H22	127.00
C4—C9—C10	109.10 (11)	C22—C23—H23	126.00
C4—C9—C11	109.41 (10)	C24—C23—H23	126.00
N1—C1—H1	126.00	C25—C26—H26	126.00
C2—C1—H1	126.00	C27—C26—H26	126.00
C3—C2—H2	126.00	C26—C27—H27	126.00
C1—C2—H2	126.00	C28—C27—H27	126.00
C4—C3—H3	126.00	N22—C28—H28	126.00
C2—C3—H3	126.00	C27—C28—H28	126.00
C7—C6—H6	126.00	C29—C30—H30A	109.00
C5—C6—H6	126.00	C29—C30—H30B	109.00
C6—C7—H7	126.00	C29—C30—H30C	109.00
C8—C7—H7	126.00	H30A—C30—H30B	110.00
N2—C8—H8	126.00	H30A—C30—H30C	109.00
C7—C8—H8	126.00	H30B—C30—H30C	109.00
C9—C10—H10A	109.00	C29—C31—H31A	109.00
C9—C10—H10B	109.00	C29—C31—H31B	109.00
C9—C10—H10C	109.00	C29—C31—H31C	109.00
H10B—C10—H10C	110.00	H31A—C31—H31B	109.00
H10A—C10—H10C	109.00	H31A—C31—H31C	110.00
H10A—C10—H10B	109.00	H31B—C31—H31C	109.00
			
C4—N1—C1—C2	0.14 (14)	N2—C5—C9—C10	170.37 (11)
C1—N1—C4—C3	−0.25 (13)	N2—C5—C9—C11	−70.77 (14)
C1—N1—C4—C9	176.69 (11)	C6—C5—C9—C10	−14.94 (19)
C8—N2—C5—C6	0.67 (14)	C6—C5—C9—C11	103.92 (15)
C8—N2—C5—C9	176.51 (11)	C5—C6—C7—C8	0.07 (16)
C5—N2—C8—C7	−0.63 (14)	C6—C7—C8—N2	0.34 (15)
C21—N21—C24—C29	−177.09 (10)	N21—C21—C22—C23	−0.10 (15)
C24—N21—C21—C22	−0.03 (13)	C21—C22—C23—C24	0.19 (15)
C21—N21—C24—C23	0.15 (13)	C22—C23—C24—N21	−0.21 (14)
C28—N22—C25—C26	−0.63 (13)	C22—C23—C24—C29	176.64 (12)
C28—N22—C25—C29	−177.43 (10)	N21—C24—C29—C25	−63.67 (14)
C25—N22—C28—C27	0.54 (14)	N21—C24—C29—C30	56.63 (15)
N1—C1—C2—C3	0.02 (14)	N21—C24—C29—C31	175.64 (11)
C1—C2—C3—C4	−0.17 (15)	C23—C24—C29—C25	119.88 (14)
C2—C3—C4—C9	−176.31 (12)	C23—C24—C29—C30	−119.82 (14)
C2—C3—C4—N1	0.25 (14)	C23—C24—C29—C31	−0.82 (18)
N1—C4—C9—C5	60.57 (15)	N22—C25—C26—C27	0.48 (13)
N1—C4—C9—C11	−179.26 (11)	C29—C25—C26—C27	176.83 (12)
C3—C4—C9—C5	−123.33 (14)	N22—C25—C29—C24	−47.82 (14)
N1—C4—C9—C10	−59.95 (15)	N22—C25—C29—C30	−168.30 (10)
C3—C4—C9—C11	−3.15 (19)	N22—C25—C29—C31	72.79 (14)
C3—C4—C9—C10	116.16 (15)	C26—C25—C29—C24	136.28 (13)
N2—C5—C9—C4	49.85 (15)	C26—C25—C29—C30	15.80 (17)
C9—C5—C6—C7	−175.73 (12)	C26—C25—C29—C31	−103.11 (15)
N2—C5—C6—C7	−0.44 (14)	C25—C26—C27—C28	−0.16 (14)
C6—C5—C9—C4	−135.46 (13)	C26—C27—C28—N22	−0.23 (14)

### Table 1 Geometry of N—H···π interactions (Å, °)

**Table:**

*D*	H	*Centroid*	N—H	H···*Cg*	*D*···*Cg*	N—H···*Cg*
N1	H1N	*Cg*4	0.86 (2)	2.534 (17)	3.2190 (12)	137.4 (14)
N2	H2N	*Cg*3	0.86 (2)	2.591 (17)	3.2425 (12)	133.7 (13)
N21	H21N	*Cg*1	0.88 (2)	2.523 (16)	3.1925 (12)	133.9 (12)
N22	H22N	*Cg*2	0.86 (2)	2.610 (17)	3.2440 (12)	131.3 (13)

*Cg*1, *Cg*2, *Cg*3 and *Cg*4 are the centroids of the N1/C1–C4, N2/C5–C8, N21/C21-C24 and N22/C25–C28 rings, respectively.
